# A novel approach to managing uncertainty in risk assessment using integrated z-numbers and intuitionistic fuzzy sets: A case study on LPG spherical tanks

**DOI:** 10.1371/journal.pone.0338798

**Published:** 2026-02-26

**Authors:** Mostafa Mirzaei Aliabadi, Vahid Ahmadi Moshiran, Omid Kalatpour, Omran Ahmadi

**Affiliations:** 1 Center of Excellence for Occupational Health, Occupational Health and Safety Research Center, School of Public Health, Hamadan University of Medical Sciences, Hamadan, Iran; 2 Department of Occupational Health and Safety Engineering, School of Public Health, Zanjan University of Medical Sciences, Zanjan, Iran; 3 Department of Occupational Health and Safety, Faculty of Medical Science, Tarbiat Modares University, Tehran, Iran; Universita degli Studi del Molise, ITALY

## Abstract

Accurate risk assessment in industrial systems is frequently challenged by uncertainty in expert judgments and system behavior. This study proposes a novel approach that integrates Intuitionistic Z-Numbers (IZNs) with the System Hazard Identification, Prediction, and Prevention (SHIPP) methodology to improve predictive reliability. IZNs capture both expert estimations and the associated confidence levels when determining the prior probabilities of basic events (BEs), thereby reducing uncertainty more effectively than conventional intuitionistic fuzzy sets. These enhanced estimates are incorporated into the SHIPP framework—utilizing fault and event tree analyses—and updated with real-world incident data via Bayesian inference. A case study involving liquefied petroleum gas (LPG) spherical storage tanks illustrates the practical application of the proposed method. Results indicate that the use of IZNs enhances the reliability of posterior probability estimates for barrier failures and associated consequences, ultimately supporting more informed and resilient risk management decisions in high-hazard industries.

## 1. Introduction

The process industry plays a crucial role in global economic development through the production of fuels, chemicals, and raw materials [1]. Within these industries, spherical storage tanks are widely regarded as a safe and efficient option due to their geometric advantages [2]. Nevertheless, a number of severe accidents involving spherical tanks have occurred in recent decades, underscoring the significant risks they pose [3]. Notable incidents include the 1984 explosion in Mexico City [4,5], the 1978 overpressure rupture at the Texas City refinery [6], the 2011 accident at the Chiba refinery in Japan [7,8], and the 1966 Feyzin refinery disaster in France [6,9]. These events emphasize the urgent need for comprehensive safety measures and accurate risk assessments in the design, operation, and maintenance of spherical storage tanks across industrial facilities [10].

All industrial processes undergo degradation over time due to a variety of factors. To ensure accurate risk estimation, risk assessment methodologies must account for these changes [1,11]. According to Khan et al. (2016), dynamic risk assessment (DRA) involves continuously updating initial risk estimates based on factors such as the performance of control systems and safety barriers, the effectiveness of maintenance and inspection activities, and the rigor of enforcement procedures [12].

Several recent studies have focused on dynamic risk assessment [13,14]. For example, Kamil et al. (2019) proposed a Petri net-based approach to evaluate the dynamic risk of domino effects, enabling the estimation of accident probabilities and spread patterns [15]. Ahmadi et al. (2020) introduced a dynamic risk assessment methodology that integrates Bayesian networks with a fuzzy Analytic Hierarchy Process (AHP) and a fuzzy Decision-Making Trial and Evaluation Laboratory (DEMATEL). This approach was used to identify and categorize safety barriers, basic events (BEs), and risk impact factors (RIFs) [16].

Rathnayaka et al. (2011) introduced the System Hazard Identification, Prediction, and Prevention (SHIPP) method, which focuses on accident modeling through the concept of safety barriers and adopts a proactive approach to risk management [3]. SHIPP is a systematic methodology designed to identify, evaluate, and predict potential accidents, with the goal of preventing their occurrence. Unlike traditional models, it incorporates the dynamic updating of accident probabilities to reflect changes over time. The method utilizes separate fault trees (FTs) for each safety barrier that mitigates the severity of consequences within an event tree (ET) framework. By incorporating Bayesian theory, SHIPP further reduces uncertainty in event prediction, thereby enhancing the accuracy of accident forecasting [17]. This methodology has been applied in several studies aimed at conducting dynamic risk assessments.

Pouyakian et al. (2021) applied the SHIPP methodology alongside the Hazard and Operability study (HAZOP) technique to reduce uncertainty related to the release of hazardous substances from floating roof tanks [18]. Similarly, other researchers have combined SHIPP with historical accident data from liquefied natural gas (LNG) facilities to estimate the posterior probability of accidents occurring within a defined timeframe [17].

Although Bayesian theory, as an integral part of the SHIPP methodology, contributes to reducing uncertainty in risk analysis, the application of specific types of fuzzy logic can have an even greater impact in producing more confident and realistic results. Experts often express their judgments about the likelihood of critical events using natural language; however, such information is frequently vague and only partially reliable, leading to increased uncertainty in the assessment process [19]. Zadeh introduced the notion of a fuzzy set (FS) to illustrate the ambiguity of information [20]. Building on this foundation, Atanassov proposed the Intuitionistic Fuzzy Set (IFS), which incorporates a degree of hesitation alongside membership and non-membership values [21]. These values quantify the extent to which an element belongs—or does not belong—to a fuzzy set, while the hesitation degree captures the uncertainty in this assessment. Compared to classical fuzzy sets, IFS provides greater flexibility for modeling and managing uncertainty [22].

Z-number, like intuitionistic fuzzy numbers, is a type of fuzzy number designed to address uncertainty in expert judgments. A Z-number consists of two components: a *limitation* component, which expresses a constraint or estimate, and a *reliability* component, which reflects the confidence level in that estimate. When intuitionistic fuzzy sets are combined with Z-numbers, they form a more advanced structure known as intuitionistic Z-numbers (IZNs). The key advantage of IZNs over Zadeh’s original Z-numbers is their improved ability to handle uncertainty. This is because both components of the Z-number are represented using membership and non-membership functions, allowing for the explicit modeling of hesitation in expert assessments [23].

Hakim et al. (2022) addressed the challenge of supplier selection by integrating Z-numbers with an intuitionistic fuzzy approach. Their findings highlighted the critical role of Intuitionistic Z-Numbers (IZNs) in capturing uncertainty within decision-makers’ assessments during complex decision-making processes [22]. In a separate study, Yuxuan Xing (2022) developed the all-Process Hydrogen Accident Risk Assessment (PHARA) model, which combines event tree analysis, intuitionistic fuzzy logic, Bayesian network analysis, HyRAM, and the Probit model to assess hydrogen-related accident risks [24]. Additionally, Dhiman et al. (2022) applied IZN to medical data—such as blood sugar levels, pulse rate, and age—for the diagnosis of dengue fever. Their results demonstrated that IZN provided more realistic and reliable outcomes compared to conventional methods [25].

Although fuzzy and probabilistic approaches have become popular in dynamic risk assessment, a clear research gap remains in integrating Intuitionistic Z-Numbers (IZNs) with established frameworks such as SHIPP. While SHIPP provides a structured methodology for modeling system hazards over time, it relies heavily on expert judgments, which are often uncertain or imprecise. Previous studies have not yet applied IZNs within SHIPP to explicitly capture both the hesitation and confidence inherent in expert assessments. Accordingly, the main objective of this study is to enhance dynamic risk assessment by integrating Intuitionistic Z-Numbers (IZNs) into the SHIPP methodology. By combining the structured framework of SHIPP with the expressive capacity of IZNs, the proposed approach aims to provide a more reliable representation of uncertainty in expert judgments, thereby improving the accuracy of failure probability estimation and supporting more informed safety decisions in high-hazard industries.

## 2. Preliminaries

### 2.1. Intuitionistic fuzzy set

The theory of Intuitionistic Fuzzy Sets (IFS) is an expansion of fuzzy set theory that enables the representation of the uncertain nature of membership, non-membership, and hesitation associated with fuzzy concepts [26].

***Definition 1*.** Let X denote a universal set and consider the Intuitionistic Fuzzy Set (IFS) denoted by $\tilde{A}$ in X, which can be expressed as [27,28].


$$\tilde{A}=\left\{\left\langle \text{x},\;{\;\mu }_{\tilde{A}}(\text{x}),\;\nu_{\tilde{A}}(\text{x})\;\right\rangle :\text{x}\in \text{X}\right\}$$ (1)

Where the membership function, ${\;\mu }_{\tilde{A}}(\text{x}\in [\text{0},\text{1}]$, and the non-membership function, $\nu_{\tilde{A}}(\text{x})\in [\text{0},\text{1}]\;$, adhere to the following condition.


$$\text{0}\leq {\;\mu }_{\tilde{A}}(\text{x})+\nu_{\tilde{A}}(\text{x})\leq \text{1}\;,\;\forall x\in X$$ (2)

The following is a definition of the hesitation degree of $x\in \tilde{A}$:


$$\pi_{\tilde{A}}(\text{x})\;=\;\text{1}-{\;\mu }_{\tilde{A}}(\text{x})-\nu_{\tilde{A}}(\text{x})$$ (3)

The IFS converts into a normal fuzzy set if $\pi_{\tilde{A}}$(x) = 0, ∀x∈X.

***Definition 2*.** If an IFS can be described as follows in terms of membership and non-membership values, then it is IF convex [29].

The membership value ${\;\mu }_{\tilde{A}}(\text{x})$ of $\tilde{A}$ exhibits a property known as intuitionistic fuzzy convexity.


$$\mu_{Ã}\left(\theta x_{1}+\left(1-\theta \right)x_{2}\right)\geq \text{min}\left(\mu_{Ã}\left(x_{1}\right),\;\;\mu_{Ã}\left(x_{2}\right)\right)\forall x\;\text{and}\;x_{2}\in x_{1},\;\theta \in \left[0,\;1\right]$$ (4)

The non-membership value $\nu_{\tilde{A}}(\text{x})$ of $\tilde{A}$ exhibits a property known as intuitionistic fuzzy convexity.


$$\nu_{Ã}\left(\theta x_{1}+\left(1-\theta \right)x_{2}\right)\geq \min\left(\nu_{Ã}\left(x_{1}\right),\;\;\nu_{Ã}\left(x_{2}\right)\right)\forall x\;\mathrm{and}\;x_{2}\in x_{1},\;\theta \in \left[0,\;1\right]$$ (5)

***Definition 3*.** An Intuitionistic Fuzzy Set (IFS) Ã in X is said to be normalized if there exist at least two points x₁, x₂ ∈ X such that the membership function ${\;\mu }_{\tilde{A}}(\text{x})$ attains the value 1 at x₁ and the non-membership function $\nu_{\tilde{A}}(\text{x})$ attains the value 1 at x₂. This ensures that the IFS includes elements with full membership and elements with full non-membership, covering the entire range of possible membership and non-membership values [30].

***Definition 4*.** An IFS $\tilde{A}=\left\{\left\langle \text{x},\;{\;\mu }_{\tilde{A}}(\text{x}),\;\nu_{\tilde{A}}(\text{x}\right\rangle :\text{x}\in \text{R}\right\}$ is referred to as an IFN when it satisfies the following criterion [28].

1) $\tilde{A}$ is IF-normal and IF-convex.
2) ${\;\mu }_{\tilde{A}}(\text{x})$ and $\nu_{\tilde{A}}(\text{x})$ are an upper and a lower semi-continuous, respectively.
3) Supp $\tilde{A}=\left\{\text{x}\in \text{X }:\nu_{\tilde{A}}(\text{x})<\text{1}\right\}$ is bounded.

A Triangular-IFN refers to an IFN that is defined in the following manner:


$${\;\mu }_{\tilde{A}}(\text{x})=\left\{ \;\begin{array}{c} @r\frac{x-a}{b-a}\;,\;a\leq x\leq b\\ \frac{c-x}{c-b}\;,\;b\leq x\leq c\;\\ \text{0}\;,\;otherwise \end{array}\right.\;$$ (6)

$${\;\nu }_{\tilde{A}}(\text{x})=\left\{ \;\begin{array}{c} @l\frac{x-a^{\prime }}{b-a^{\prime }}\;,\;a^{\prime }\leq x\leq b\\ \frac{c^{\prime }-x}{c^{\prime }-b}\;,\;b\leq x\leq c^{\prime }\;\\ \;\text{0}\;,\;otherwise \end{array}\right.\;$$ (7)

Where $a^{\prime }\leq a\leq b\leq c\leq c^{\prime }$. Then triangular IFN is denoted by $\tilde{A}$ = (a, b, c; $a^{\prime },\;b,\;c^{\prime }$).

***Definition 5.*** Let $\;\tilde{A}$ = ([$a_{\text{1}},a_{\text{2}},a_{\text{3}}$]; ${\;\mu }_{\tilde{A}}(\text{x}),\;\nu_{\tilde{A}}(\text{x})$) and $\tilde{B}$ = ([$b_{\text{1}},b_{\text{2}},b_{\text{3}}$]; ${\;\mu }_{\tilde{B}}(\text{x}),\;\nu_{\tilde{B}}(\text{x})$) be two positives triangular IFNs (TIFN). Furthermore, y is the real number and y≥0. The following is the definition of the TIFN sets’ main operations [31,32].


$$\tilde{A}+\tilde{B}=\left(\left[a_{\text{1}}+b_{\text{1}},a_{\text{2}}+b_{\text{2}},\;a_{\text{3}}+b_{\text{3}}\right];\text{min}\left(\mu_{\tilde{A}}(\text{x}),{\;\mu }_{\tilde{B}}(\text{x})\right),\text{max}\left(\nu_{\tilde{A}}(\text{x}),\nu_{\tilde{B}}(\text{x})\right)\right)$$ (8)

$$\tilde{A}-\tilde{B}=\left(\left[a_{\text{1}}-b_{\text{1}},a_{\text{2}}-b_{\text{2}},\;a_{\text{3}}-b_{\text{3}}\right];\text{min}\left(\mu_{\tilde{A}}(\text{x}),{\;\mu }_{\tilde{B}}(\text{x})\right),\text{max}\left(\nu_{\tilde{A}}(\text{x}),\nu_{\tilde{B}}(\text{x})\right)\right)\;$$ (9)

$$\tilde{A}.\tilde{B}=\left(\left[a_{\text{1}}.b_{\text{1}},a_{\text{2}}.b_{\text{2}},\;a_{\text{3}}.b_{\text{3}}\right];\text{min}\left(\mu_{\tilde{A}}(\text{x}),{\;\mu }_{\tilde{B}}(\text{x})\right),\text{max}\left(\nu_{\tilde{A}}(\text{x}),\nu_{\tilde{B}}(\text{x})\right)\right)$$ (10)

$$y.\tilde{A}=\left(\left[y.a_{\text{1}},y{.a}_{\text{2}},y.a_{\text{3}}\right];{\;\mu }_{\tilde{A}}(\text{x}),\nu_{\tilde{A}}(\text{x})\right)$$ (11)

### 2.2. Z-number

**Fig 1:**
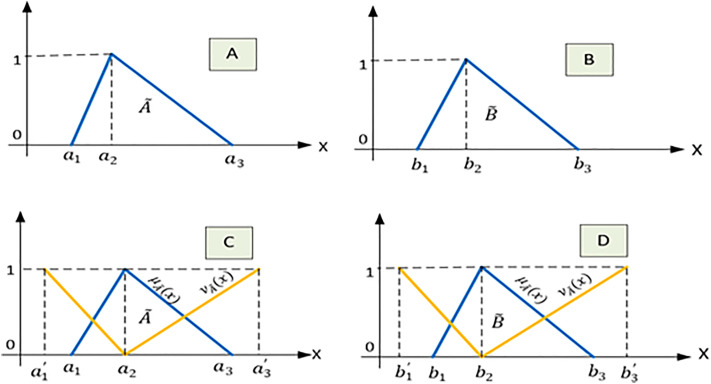
Comparison of fuzzy Z-number and intuitionistic fuzzy Z-number. A and B: a simple fuzzy Z-number. C and D: a simple intuitionistic fuzzy Z-number.


The concept of Z-numbers was introduced by Zadeh (2011) [33]. A Z-number, represented as Z = ($\tilde{A}$, $\tilde{B}$), comprises two ordered fuzzy components. The first component, A, assesses parameter values under uncertain circumstances. The second component, B, quantifies the reliability or degree of certainty associated with A. Fig 1(A and B) illustrates the parts $\tilde{A}$ and $\tilde{B}$ of a simple Z-number [34]. Usually, $\tilde{A}$ and $\tilde{B}$ are based on personal opinions and can be expressed using Simple words [35]. Eq. 12 provides the restriction function ˝R(X): X is A˝ [36].

$$\text{R}(\text{X}):\text{ X is A}\to \text{ Poss }(\text{X }=\text{ x})\;={\;\mu }_{\tilde{A}}(\text{x})\;$$ (12)

***Definition 6*.** Consider Z = ($\tilde{A}$, $\tilde{B}$) as a z-number, where $\tilde{A}$ = {(x, ${\;\mu }_{\tilde{A}}(\text{x})$) | x∈[0, 1] } and $\tilde{B}$ = {(x, ${\;\mu }_{\tilde{B}}(\text{x})$) | x∈[0, 1] } represent triangular membership functions. Equations (9) to (10) can be employed to calculate a Z-number’s corresponding regular fuzzy number [34].

The certainty is expressed as a definite number using Eq. 13.


$$\alpha =\frac{\int x\mu_{\tilde{B}}\;(x)dx}{\int \mu_{\tilde{B}}\;(x)dx}$$ (13)

By considering α as the weight of the second portion $\tilde{B}$ and $\mu_{\tilde{B}}\;(x)$ as the measure of x ∈ X’s dependency on B, Eq. 14 enables the addition of α to $\tilde{A}$.


$$\tilde{\text{Z}}\mathrm{\backslash vspace}{0.5mm}^{\alpha }=\left\{\;\left(\text{x},\;\mu_{{\tilde{\text{A}}}^{\alpha }}\;(\text{x})\right)|\mu_{{\tilde{\text{A}}}^{\alpha }}(\text{x})=\alpha \mu_{\tilde{\text{A}}}(\text{x}),\;\mu (\text{x})\in (\text{0},\;\text{1})\right\}$$ (14)

The conversion process of a weighted Z-number into a type-1 fuzzy number, denoted as ${\tilde{Z}}^{\prime }$, is accomplished by employing Eq. 15.


$${\tilde{Z}}^{\prime }=\left\{\;\;\;\;\;\left(x,\;\mu_{{\tilde{Z}}^{\prime }}(x)\right)|\mu_{{\tilde{Z}}^{\prime }}(x)=\mu_{\tilde{A}}\left(\frac{x}{\sqrt{\alpha }}\right),\mu (x)\in [\text{0},\text{1}\;\;\right\}$$ (15)

### 2.3. Intuitionistic Z-number (IZN)

***Definition 7.*** Intuitionistic fuzzy Z-number is characterized by an ordered pair of fuzzy sets, denoted as Z = ($\tilde{A}$, $\tilde{B}$), where the $\tilde{A}$, and $\tilde{B}$ are represented as fallows [37].


$$\tilde{A}=\left\{\left\langle x,\mu_{\tilde{A}},\nu_{\tilde{A}}|x\in \;X\right\rangle \right\}=\left(a_{\text{1}},a_{\text{2}},a_{\text{3}};\;a_{\text{1}}^{\prime },a_{\text{2}}\;,a_{\text{3}}^{\prime }\;\right)$$ (16)

$$\tilde{B}=\left\{\left\langle x,\mu_{\tilde{B}},\nu_{\tilde{B}}|x\in \;X\right\rangle \right\}=\left(b_{\text{1}},b_{\text{2}},b_{\text{3}};\;b_{\text{1}}^{\prime },b_{\text{2}}\;,b_{\text{3}}^{\prime }\;\right)$$ (17)

Figs 1C and 1D visually represent IZN.

## 3. The proposed method

This section outlines the implementation steps of the proposed method based on the SHIPP approach. The SHIPP methodology is structured into four phases: system definition, risk identification and analysis, accident prediction, and the implementation of accident prevention strategies [38]. Given that the primary objective of this study is to reduce uncertainty in risk assessment, the focus is placed on the first three phases, which inherently contribute to improvements in the fourth phase.

**Fig 2:**
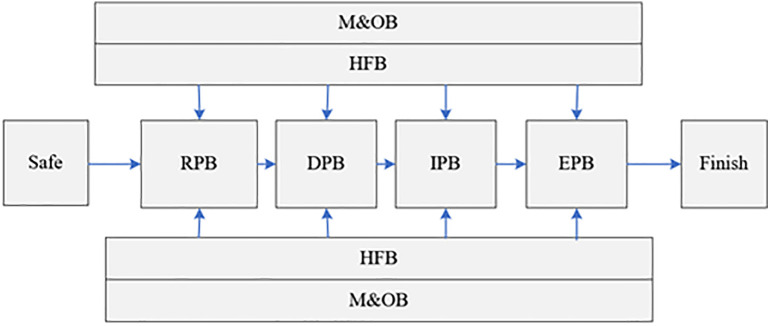
The process accident model [38].

In the system definition phase, safety barriers are identified according to the SHIPP framework and categorized into six groups: Release Prevention Barrier (RPB), Dispersion Prevention Barrier (DPB), Ignition Prevention Barrier (IPB), Escalation Prevention Barrier (EPB), Human Factor Barrier (HFB), and Management and Organizational Barrier (M&OB), as illustrated in Fig 2 [38].

In the analysis phase, fault trees (FTs) were developed for each of the primary barriers identified, in accordance with the SHIPP methodology. These fault trees were constructed based on credible references and prior research studies [2,17,35,39,40]. Section 3.2 details the methodology used to calculate the probability of failure for each barrier using fault tree analysis, as well as the arrangement of barriers to prevent the occurrence of adverse consequences.

The dataset used in this research consisted of two main parts. The first part included the prior probabilities of basic events in the barrier fault tree, which were obtained from expert judgments using linguistic terms. The procedures for eliciting, quantifying, integrating, and aggregating expert opinions—along with their associated confidence levels—are thoroughly explained in Sections 3.1 to 3.1.5. To collect these data, the fault tree was presented on slides, and the experts, while observing the overall structure of the tree and the interrelationships between different events, expressed their opinions regarding the prior probability of each basic event. The second part comprised accident and incident records of the selected equipment during the past 12 months, collected from official reports and documentation, and further explained in Section 4.3.

**Fig 3:**
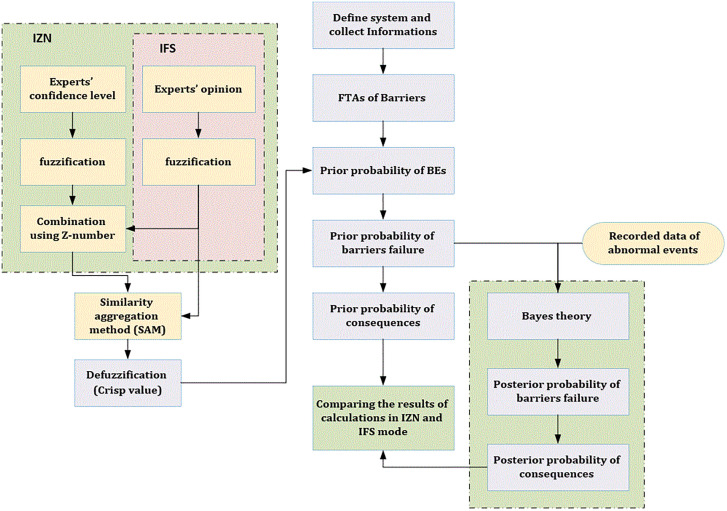
Framework of the proposed method.

The third phase of the SHIPP approach involves predicting the likelihood of accidents over a defined future time horizon. The calculations relevant to this stage are described in detail in Section 3.3. An overview of the entire framework proposed in this study is illustrated in Fig 3.

### 3.1. Employing the expert judgment

In this section, six experts provide individual assessments of the likelihood of BEs occurring. Given the differences in their expertise, academic background, and professional experience, variability in their evaluations is expected. To address this, a weighting system—outlined in Table 1—is employed to assign appropriate weights to each expert’s opinion. This approach ensures that the aggregated assessments reflect the relative credibility and qualifications of the contributors, thereby enhancing the reliability of the overall evaluation.

**Table 1:** Weighting scores and details related to experts [41].

Group	Classification	Score
Job title	Operator	1
	Technical	2
	Engineer	3
	Manager, Factory inspection	4
	Chief Engineer, Director	5
Educational degree	High school	1
	Higher national diploma	2
	Bachelor	3
	Master	4
	PhD	5
Work experience (year)	≤ 5	1
	6 to 9	2
	10 to 19	3
	20 to 29	4
	≥ 30	5

#### 3.1.1. Quantification of linguistic terms.

Experts express their evaluations of the likelihood of BEs using linguistic terms, which serve as qualitative descriptors. To enable further analysis and computation in the subsequent risk assessment and prediction stages, these linguistic terms must be translated into quantitative values. Table 2 presents the corresponding Intuitionistic Triangular Fuzzy Numbers (TIFNs) used to represent both expert opinions and their associated confidence levels. The following step outlines the procedure for combining these intuitionistic fuzzy sets to capture both dimensions in a unified representation.

**Table 2:** Linguistic terms and corresponding IFS [19,41].

Linguistic terms of opinion	Intuitionistic triangular fuzzy sets	Linguistic terms of confidence level	Intuitionistic triangular fuzzy sets
Very Low (VL)	(0.00, 0.04, 0.08; 0.00, 0.04, 0.08)	Not sure (NS)	(0.00, 0.20, 0.40; 0.00, 0.20, 0.40)
Low (L)	(0.07, 0.13, 0.19; 0.06, 0.13, 0.20)	Relatively sure (RS)	(0.20, 0.40, 0.60; 0.10, 0.40, 0.70)
Medium Low (ML)	(0.17, 0.27, 0.37; 0.15, 0.27, 0.39)	Sure (S)	(0.40, 0.60, 0.80; 0.30, 0.60, 0.90)
Medium (M)	(0.35, 0.50, 0.65; 0.32, 0.50, 0.68)	Very Sure (VS)	(0.60, 0.80, 1.00; 0.60, 0.80, 1.00)
Medium High (MH)	(0.62, 0.73, 0.82; 0.61, 0.73, 0.85)		
High (H)	(0.81, 0.87, 0.93; 0.79, 0.87, 0.95)		
Very High (VH)	(0.92, 0.96, 1.00; 0.92, 0.96, 1.00)		

#### 3.1.2. Regular fuzzy number.

Due to failure probability represented by the IZN that has TIFN in both parts A and B, denoted as Z = {$\left(a_{\text{1}},a_{\text{2}},a_{\text{3}};\;a_{\text{1}}^{\prime },a_{\text{2}}\;,a_{\text{3}}^{\prime }\;\right),(b_{\text{1}},b_{\text{2}},b_{\text{3}};\;b_{\text{1}}^{\prime },b_{\text{2}}\;,b_{\text{3}}^{\prime })$}, the following steps can be applied to achieve defuzzification and obtain the crisp value [26,42].

First, part B of Z -number, $\left(b_{\text{1}},b_{\text{2}},b_{\text{3}};\;b_{\text{1}}^{\prime },b_{\text{2}}\;,b_{\text{3}}^{\prime }\right)$, defuzzifide by Eq. 18 to obtain crisp possibility score (S) [42].


$$S=\frac{\text{1}}{\text{3}}[\frac{{(b}_{\text{3}}^{\prime }-b_{\text{1}}^{\prime })(b_{\text{2}}-\text{2}b_{\text{3}}^{\prime }-\text{2}b_{\text{1}}^{\prime })+(b_{\text{3}}-b_{\text{1}})(b_{\text{1}}+b_{\text{2}}+b_{\text{3}})+\text{3}({b_{\text{3}}^{\prime }}^{\text{2}}-{b_{\text{1}}^{\prime }}^{\text{2}})}{b_{\text{3}}^{\prime }-b_{\text{1}}^{\prime }+b_{\text{3}}-b_{\text{1}}}]$$ (18)

Since the elements in the denominator correspond to the linguistic terms for confidence levels, and given the triangular intuitionistic fuzzy values presented in the confidence level section of Table 2, it is evident that the denominator of the fraction cannot be zero.

Second, the regular fuzzy number was obtained using multiplying the crisp possibility score of part B by part A (Eq. 19).


$${\;\tilde{Z}}^{\alpha }=\left(\tilde{\tilde{A}},\;\sqrt{S_{B}}\right)\to {\tilde{Z}}^{\prime }=\left(\sqrt{S_{B}}\times \tilde{\tilde{A}}\right)\;\overset{Eq.\text{1}\text{1}}{\to }\;{\tilde{Z}}^{\prime }=(\sqrt{S_{b\text{1}}}\;{\times a}_{\text{1}},\;\sqrt{S_{b\text{2}}}\;{\times a}_{\text{2}},\;\sqrt{S_{b\text{3}}}\;{\times a}_{\text{3}};\;\sqrt{S_{b_{\text{1}}^{\prime }}}\;\times a_{\text{1}}^{\prime },\;\sqrt{S_{b\text{2}}}\;{\times a}_{\text{2}},\;\sqrt{S_{b_{\text{3}}^{\prime }}}\;{\times a}_{\text{3}}^{\prime })$$ (19)

#### 3.1.3. Experts’ opinion aggregation.

To achieve consensus among experts with diverse backgrounds and perspectives, it is essential to aggregate their individual evaluations into a single, representative numerical value. This is accomplished using the Similarity Aggregation Method (SAM). The following subsection outlines the step-by-step procedure of the SAM approach [43].

***Step1. Calculating the similarity degree:*** The similarity measure between the opinions of ${\tilde{A}}_{i}$ and ${\tilde{A}}_{j}$ put forth by experts $E_{i}$ and $E_{j}$ can be determined by utilizing the similarity measure function $S_{ij}\left({\tilde{A}}_{i},\;{\tilde{A}}_{j}\right)$. The following definition characterizes this function.


$$S_{ij}\left({\tilde{A}}_{i},\;{\tilde{A}}_{j}\right)=\left\{ \begin{array}{c} @r\frac{{EV}_{i}}{{EV}_{j}}\;,\;if\;{EV}_{i}\leq {EV}_{j}\;\\ \frac{{EV}_{j}}{{EV}_{i}}\;,\;if\;{EV}_{j}\leq {EV}_{i\;} \end{array}\right.\;$$ (20)

The function $S_{ij}\left({\tilde{A}}_{i},\;{\tilde{A}}_{j}\right)$, which yields a value between 0 and 1, serves as the similarity measure for comparing two standard intuitionistic fuzzy numbers, ${\tilde{A}}_{i}$ and $\;{\tilde{A}}_{j}$. The variables ${EV}_{i}$ and ${EV}_{j}$ represent the expectancy evaluation of ${\tilde{A}}_{i}$ and $\;{\tilde{A}}_{j}$, respectively.

The expectancy evaluation of TIFN $\tilde{A}=\left(a_{\text{1}},a_{\text{2}},a_{\text{3}};\;a_{\text{1}}^{\prime },a_{\text{2}}\;,a_{\text{3}}^{\prime }\;\right)$ calculation is as follows:


$$EV\left(\tilde{A}\right)=\frac{\left(a_{\text{1}}+a_{\text{1}}^{\prime }\right)+\text{4}\times b+(a_{\text{3}}+a_{\text{3}}^{\prime })}{\text{8}}$$ (21)

The similarity matrix (SM) for a total of n experts is formally defined as:


$$SM=[\begin{array}{ccc} \text{1} & s_{\text{1}\text{2}} & \begin{array}{ccc} s_{\text{1}\text{3}} & \cdots & s_{\text{1}n} \end{array}\\ s_{\text{2}\text{1}} & \text{1} & \begin{array}{ccc} s_{\text{2}\text{3}} & \cdots & s_{\text{2}n} \end{array}\\ \begin{array}{c} \vdots \\ s_{n\text{1}} \end{array} & \begin{array}{c} \vdots \\ s_{n\text{2}} \end{array} & \begin{array}{ccc} \begin{array}{c} \vdots \\ s_{n\text{3}} \end{array} & \begin{array}{c} \ddots \\ \cdots \end{array} & \begin{array}{c} \vdots \\ \text{1} \end{array} \end{array} \end{array}]$$ (22)

Where $s_{ij}=S_{ij}\left({\tilde{A}}_{i},\;{\tilde{A}}_{j}\right)$ if i=j then $s_{ij}=\text{1}.$

**Step2. Calculating the degree of agreement**: The Eq. 23 is used to find the average agreement degree $AA\left(E_{i}\right)$ for each expert $E_{i}$:


$$AA\left(E_{i}\right)=\frac{\text{1}}{n-\text{1}}\sum {\mathrm{\backslash nolimits}}_{\;\;\;\begin{array}{c} j=j\\ j\neq \text{1} \end{array}}^{n}S_{ij}\left({\tilde{A}}_{i},\;{\tilde{A}}_{j}\right)\;i=\text{1},\;\text{2},\;\ldots ,\;n.$$ (23)

***Step 3. Calculation of relative agreement****:* The following Equation is used to calculate the relative agreement degree $RA\left(E_{i}\right)$ for each expert $E_{i}$:


$$RA\left(E_{i}\right)=\frac{AA\left(E_{i}\right)}{\sum_{i=\text{1}}^{n}AA(E_{i})}\;i=\text{1},\;\text{2},\;\ldots ,\;n.$$ (24)

***Step 4. Computing the weighting factor****:* weighting factor of each expert ($\text{WF}\left({\text{E}}_{\text{i}}\right)$) is computed as follows:


$$WF\left(E_{i}\right)=\frac{WS\left(E_{i}\right)}{\sum_{i=\text{1}}^{n}WS\left(E_{i}\right)}\;i=\text{1},\;\text{2},\;\ldots ,\;n.$$ (25)

The $\text{WS}\left({\text{E}}_{\text{i}}\right)$ is the weighting score that considers the sum of each expert’s education level, job title, and work experience (Table 1).

***Step 5: Calculating consensus degree C(E***_***i***_***):*** The total weight attributed to expert E_i_ is obtained by integrating their RA and WF components.


$$C\left(E_{i}\right)=\beta ⊙\;WF\left(E_{i}\right)+(\text{1}-\beta )⊙\;RA\left(E_{i}\right)$$ (26)

Where the relaxation factor that can be applied to $WF\left(E_{i}\right)$ and $RA\left(E_{i}\right)$ to determine their relative significance is denoted by β (0 ≤ β ≤ 1). In this study, a value of 0.5 is adopted for β to achieve equal weighting of the variables.


$${\tilde{P}}_{j}=\sum {\mathrm{\backslash nolimits}}_{i=\text{1}}^{n}C\left(E_{i}\right)⊗{\tilde{P}}_{ij},\;j=\text{1},\;\text{2},\;\ldots ,\;n.$$ (27)

Where the ${\tilde{P}}_{j}$ represents the aggregation possibility of ${\text{ BE}}_{j}$ in the form of IFSs. The ${\tilde{P}}_{ij}\;$ is opinions of experts.

#### 3.1.4. Defuzzification of Intuitionistic Z-numbers (IZN).

Defuzzification is required to convert the Intuitionistic Fuzzy Failure Probability (IFFP) of a basic event (BE) into a crisp numerical value. The regular Intuitionistic Fuzzy Set (IFS) is first derived using Equations (18) and (19), as described and aggregated in Subsection 3.1.3. Subsequently, Part A of the IFS—representing expert opinion—is defuzzified in the same manner as Part B (confidence level), using Equation (18) to obtain a final crisp possibility score.

#### 3.1.5. Converting crisp possibility score (S) into failure probability (FP).

Using the following function [44], the expert-rated crisp possibility score has to be transformed into the FP:


$$FP=\left\{ \begin{array}{c} @r\frac{\text{1}}{{\text{1}\text{0}}^{K}}\;S\neq \text{0}\\ \text{0}\;S=\text{0} \end{array}\right.\;$$ (28)

$$K={\left[\frac{\text{1}-S}{S}\right]}^{\text{1}/\text{3}}\times \text{2}.\text{3}\text{0}\text{1}$$ (29)

Where “S” is the crisp possibility score.

### 3.2. Calculating the probability of events and consequences

#### 3.2.1. Fault tree (FT) of barrier failure.

Three primary techniques are available for calculating the likelihood of a top event (i.e., barrier failure): Monte Carlo simulation, the minimal cut set method, and the gate-by-gate approach. In this study, the gate-by-gate method was employed. In an AND gate configuration, all input events must occur for the output event to take place, whereas in an OR gate, the occurrence of any single input event is sufficient to trigger the output. The probabilities of intermediate events (IEs) and top events (TEs) are calculated based on the probabilities of the associated basic events (BEs), using the logic of AND and OR gates, as shown in Equations (30–31) [45]. To quantify the BEs, the Intuitionistic Fuzzy approach integrated with Z-numbers (IZN), as detailed in previous sections, was used.


$$P_{OR}=\text{1}-\prod {\mathrm{\backslash nolimits}}_{i=\text{1}}^{n}\left(\text{1}-P_{i}\right)$$ (30)

$$P_{AND}=\prod {\mathrm{\backslash nolimits}}_{i=\text{1}}^{n}P_{i}$$ (31)

$$P_{TE}=\prod {\mathrm{\backslash nolimits}}_{j\in M}\;\left(\text{1}-\prod {\mathrm{\backslash nolimits}}_{{BE}_{i\in Qj}}\;\;\left(\text{1}-P_{i}\right)\right)$$ (32)

where Pi represents the probability of occurrence of ${\text{BE}}_{i}$, and Qj represents a single BE or group of basic events in the fault tree structure.

#### 3.2.2. Event tree.

**Fig 4:**
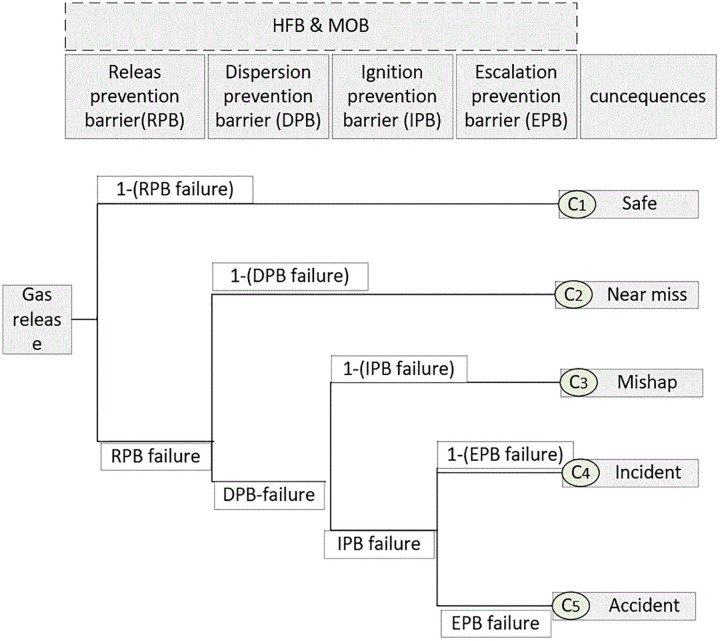
The severity of consequences following barrier failures in each level of the event tree.

According to the SHIPP methodology, the sequence of barriers designed to prevent or mitigate the severity of consequences is arranged as follows: RPB, DPB, IPB, and EPB, as illustrated in Fig 4. Each barrier in the sequence can either succeed or fail. If one barrier fails, the next is activated, progressing through a two-stage (success/failure) sequence that continues until the final barrier is reached. Additionally, two other barriers—HFB and M&OB—play an indirect role in reducing the severity of consequences by influencing the performance of the primary barriers within the operational environment. This overlap in influence contributes to the complexity of expert judgment when estimating the probabilities of underlying events.

In subsection 3.2.1, the probability of barrier failure was estimated by employing fault tree analysis uniquely tailored to each obstacle. This estimation, based on the algorithm depicted in Fig 4, was used to calculate the probability of consequence at the k (*k* = 1, 2, 3, 4, 5) level $\left(P\left(C_{k}\right)\right)$ as follows [46]:


$$P\left(C_{k}\right)=\prod {\mathrm{\backslash nolimits}}_{j\in {SB}_{k}}x_{t}^{\theta_{i\;k}}{\left(\text{1}-x_{t}\right)}^{\text{1}-\theta_{i\;k}}$$ (33)

${SB}_{k}\;$ is a safety barrier at level k. If $\theta_{i\;k}$=0, The upper branch of level k is considered (success); If $\theta_{i\;k}$=1, The down branch of level k is considered (failure).

### 3.3. Mechanism of updating

Barrier failure rates, represented as top events in the fault tree, are derived using real operational data from the industry under study. However, these failure rates are typically subject to uncertainty. To address this, Bayes’ theorem is applied to update the failure probabilities, thereby reducing uncertainty and improving the accuracy of the estimates. In theory, this Bayesian updating process yields results that more closely reflect actual system behavior.

The Bayesian equation for calculating the posterior probability of barrier failures (as top events) is as follows [47]:


$$P\left(\frac{x_{i}}{data}\right)=\frac{P\left(\frac{data}{x_{i}}\right).P\left(x_{i}\right)}{\sum p\left(\frac{data}{x_{i}}\right).P\left(x_{i}\right)}$$ (34)

Where $x_{i}$ is the failure probability of the *ith* safety. The prior probability of $x_{i}$, denoted as P($x_{i}$), represents the probability assigned to the failure probabilities of the safety barriers RPB, DPB, IPB, and EPB, refers to the initial estimate assigned to the failure likelihood of safety barriers such as RPB, DPB, IPB, and EPB. These prior probabilities are derived using the Intuitionistic Z-Number (IZN) methodology introduced earlier in the study, which improves the reliability of expert-based estimates. *data* is recorded data of abnormal events from the plant, and *p(data/xi)* is the likelihood probability of abnormal event. The denominator shows the normalization factor.

The collected plant data on abnormal events is used to compute the likelihood values *p(data/xi)* as follow:


$$p\left(\frac{data}{x_{i}}\right)=\frac{N_{f,i}}{N_{f,i}\;+\;N_{S,i}}$$ (35)

Where $N_{S,i}\;$ is the number of barrier successes and $N_{f,i}$ is the number of barrier failures that have the following relations:


$$N_{S,i}=N_{C,\;k},\;for\;k=i$$

$$N_{f,i}=\sum {\mathrm{\backslash nolimits}}_{k>i}N_{C,\;k}\;,\;for\;k>i\;;i=\text{1},\text{2},\text{3}\;and\;k=\text{1},\;\text{2}\;,\text{3},\;\text{4},\;\text{5}$$ (36)

Where $N_{C,\;k}$ is the number of consequences occurrence.

Using the posterior probability of obstacles’ failure, the consequences’ posterior probability can be calculated according to the ETA described (Eq. 33) in the previous section.

## 4. Application of methodology: A case study

The proposed method was applied in a case study at an oil refinery located in Tabriz, Iran. This refinery was selected because it operates spherical liquefied petroleum gas (LPG) storage tanks that are critical for industrial operations and represent a high-risk scenario in terms of potential accidents. The site was chosen due to the availability of detailed operational and incident data, as well as access to experienced experts familiar with the equipment and safety procedures. In this refinery, spherical storage tanks containing LPG were selected due to their critical role in storing large volumes of LPG under high pressure, which makes them highly hazardous in case of failure. LPG vapor can form an explosive mixture when its concentration in air ranges from 2% to 10% (LEL–UEL), making such leaks highly hazardous and potentially leading to fires or severe explosions [2]. This case study aims to demonstrate the practicality and effectiveness of the proposed approach in addressing uncertainty and enhancing risk prediction. The analysis includes fault tree modeling for each safety barrier, estimation of both prior and posterior probabilities, and consequence prediction using event tree analysis.

### 4.1. Fault tree of barrier failure

**Fig 5:**
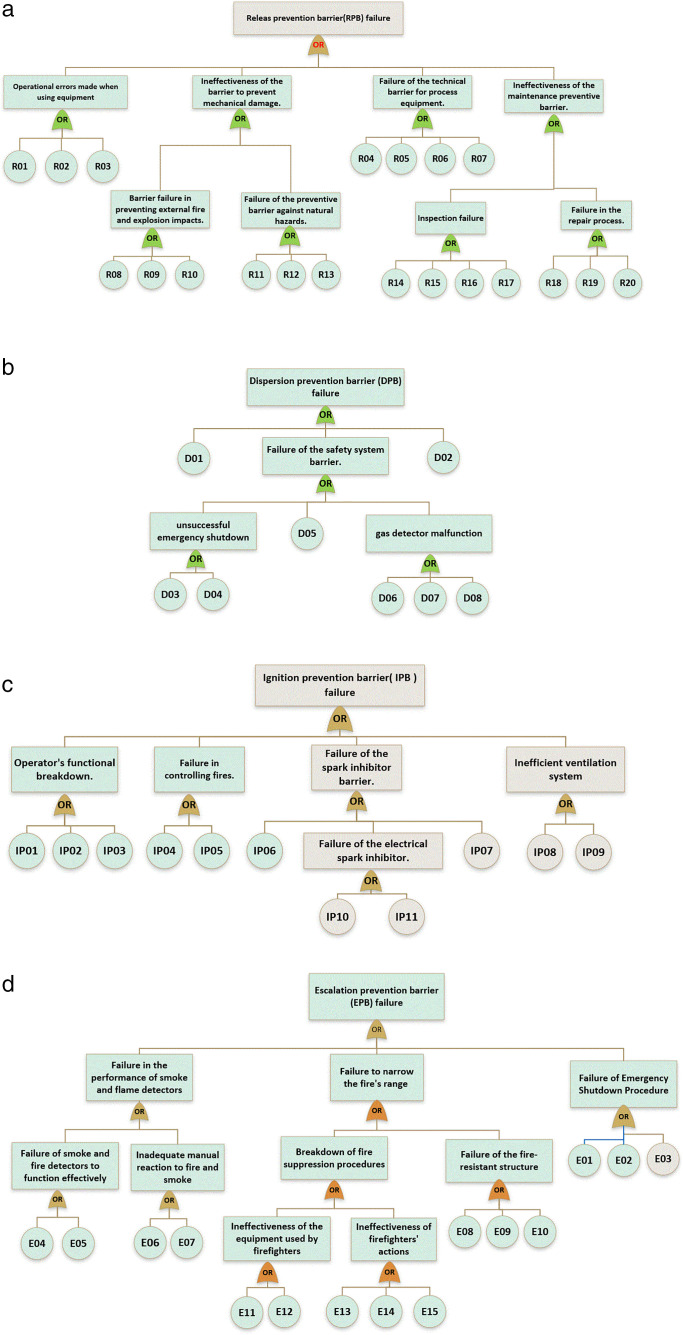
Fault Trees of Different Process Barriers. A. Fault tree of RPB. b. Fault tree of DPB.c. Fault tree of IPB.d. Fault tree of EPB.

Following the identification and preliminary analysis of the system under study, fault trees were constructed for each of the four primary safety barriers in accordance with the SHIPP methodology. These barriers include RPB, DPB, IPB, and EPB. The structures of these fault trees are illustrated in Figs 5a–5d, while Table 3 provides detailed descriptions of the corresponding Basic Events (BEs) associated with each barrier.

**Table 3:** Description of the basic events symbols.

Symbol	Event description	Symbol	Event description
R01	Error in setting desired valve level	D08	Low sensitivity of the gas sensor
R02	Valves and equipment without proper labels	IP01	Incorrect permit to work utilization.
R03	Failure to identify defects	IP02	Neglecting the prohibition of smoking sign.
R04	Malfunction of liquid level indicator	IP03	Conducting hot work without the necessary permit.
R05	Improper operation of the pressure gauge	IP04	Defective operation of the detector.
R06	Defective safety valve	IP05	Lack of a detection device.
R07	Failure of aging prevention barrier	IP06	Faulty operation of the grounding system.
R08	Firefighters’ inadequate reaction	IP07	Failure of the strike spark preventive barrier
R09	malfunction of the cooling system	IP08	Absence of ventilation equipment.
R10	Body insulation failure in spherical tanks	IP09	The ventilation system is active but inadequate.
R11	Poor tank construction	IP10	Illicit mobile device usage.
R12	structure in the tank’s vicinity that pose a threat	IP11	Unauthorized welding activity.
R13	Failure of lightning arrester	E01	Failure of a sensor linked to the emergency shutdown system.
R14	Inspectors lacking sufficient experience	E02	Malfunctioning valve for emergency shut-off.
R15	Inspectors with limited knowledge	E03	Inadequate leak sealing
R16	Absence of thorough inspection instructions	E04	Incompatibility of the detector with the type of fire
R17	Failure of the leakage test protocol	E05	faulty detector
R18	The use of substandard materials	E06	Malfunctioning manual alarm activation.
R19	Insufficient training of repairmen	E07	Operator response delay owing to alarm non-operation or misunderstanding
R20	Improper equipment installation	E08	Defective operation of the cooling system.
D01	Delayed operator response time.	E09	Insufficient barrier that resists fire
D02	Breakdown of physical barriers	E10	Non-compliance with the required gap between tanks and nearby facilities.
D03	Difficulty in reaching the manual emergency stop valve.	E11	failure of the fire suppressor (sprinkler)
D04	Failure to have a manual/Automatic shut-off valve.	E12	Firefighting apparatuses that are old and worn out.
D05	Defective operation of the emergency shut down	E13	Exhaustion of firefighters.
D06	Lack of proper detector area coverage.	E14	Improper command of firefighting teams
D07	Faulty gas monitoring device.	E15	Firemen not equipped with proper safety attire.

Basic Events (BEs) were assessed by six experts using linguistic terms for probability and confidence, which were quantified through Intuitionistic Triangular Fuzzy Numbers (TIFNs). To better handle uncertainty, these inputs were converted into Intuitionistic Z-Numbers (IZNs), combining probability and reliability, and then defuzzified into crisp values. These probabilities were used in fault trees to estimate the likelihood of safety barrier failures, which served as inputs for consequence modeling via event trees. This approach effectively incorporates expert subjectivity and system uncertainty into the risk assessment process.

### 4.2. Determining the prior probability

This study was conducted with ethical approval from Hamadan University of Medical Sciences (Research Ethics Certificate: IR.UMSHA.REC.1401.572).

To estimate the prior probability of safety barrier failure, six experts were selected. Experts were recruited and data were collected for this case study between February 1, 2024, and September 30, 2024. Table 4 summarizes their qualifications and outlines the scoring criteria used to assign weight factors.

**Table 4:** Scores and weighting factors related to experts.

Symbol	Experts	Job title	Educational degree	Work experience	Score	Weight factor (Wi)
E1	Refining Engineer	4	4	3	11	0.1964
E2	Head of HSE	4	4	3	11	0.1964
E3	HSE Expert	3	3	2	8	0.1428
E4	Chief of the fire department	4	3	3	10	0.1785
E5	Technical inspection of tanks	4	3	2	9	0.1607
E6	Process Safety Expert	3	3	1	7	0.125

Each expert was asked to evaluate the likelihood of occurrence of each BE and indicate their level of confidence in that evaluation using linguistic terms defined in Table 2. These terms captured the uncertainty inherently present in expert judgment. The detailed records of expert opinions and confidence levels for all BEs are provided in S1 Table (S1A and S1B in S1 Table).

It is important to note that these experts participated solely to support the research team in analyzing technical aspects of the industrial system and identifying potential sources of failure. No personal or identifiable data were collected, and the study was conducted in accordance with ethical guidelines approved by the university’s ethics committee. As such, verbal consent was deemed sufficient and appropriate for this form of expert elicitation.

To better capture uncertainty and expert confidence, intuitionistic fuzzy values for opinion and confidence were integrated using Z-number theory, resulting in Intuitionistic Z-Numbers (IZNs). The IZNs were then converted into regular intuitionistic fuzzy sets using Equation (19) for further analysis.

To reach a consensus estimate for each BE, the Similarity Aggregation Method (SAM) was applied (Equations 20–27). This method combines the weighted input of each expert with a similarity-based agreement measure to produce an aggregated fuzzy set. These aggregated results were then defuzzified using Equation (18) to yield crisp numerical values representing the prior failure probability of each BE. An example of the full aggregation and defuzzification process for BE R10 is provided in S1 Appendix, offering transparency and clarity in the calculation process.

The final results are presented in Table 5, which displays the prior probabilities of all BEs calculated under two different approaches:

**Table 5:** Fuzzy failure probability of BEs based in states of IFS and IZN.

BEs	IZN		IFS		BEs	IZN		IFS	
	S	FFP	S	FFP		S	FFP	S	FFP
R01	0.4463	0.0033	0.6210	0.0111	D08	0.4156	0.0026	0.5346	0.0063
R02	0.5013	0.0050	0.6392	0.0125	IP01	0.2164	0.0002	0.2878	0.0007
R03	0.4392	0.0031	0.5968	0.0095	IP02	0.3355	0.0012	0.3752	0.0018
R04	0.4736	0.0041	0.5903	0.0091	IP03	0.2507	0.0004	0.3024	0.0009
R05	0.4265	0.0028	0.5746	0.0082	IP04	0.3150	0.0010	0.3710	0.0018
R06	0.3445	0.0014	0.4317	0.0030	IP05	0.3292	0.0012	0.4987	0.0049
R07	0.4972	0.0049	0.5935	0.0093	IP06	0.3567	0.0015	0.4659	0.0039
R08	0.3524	0.0015	0.4346	0.0030	IP07	0.4624	0.0038	0.5701	0.0080
R09	0.4823	0.0044	0.6574	0.0140	IP08	0.5041	0.0051	0.6232	0.0113
R10	0.3930	0.0021	0.5408	0.0066	IP09	0.3002	0.0008	0.4380	0.0031
R11	0.4752	0.0041	0.5906	0.0092	IP10	0.3316	0.0012	0.4461	0.0033
R12	0.4336	0.0030	0.6431	0.0128	IP11	0.3850	0.0020	0.5202	0.0057
R13	0.3334	0.0012	0.4218	0.0027	E01	0.2251	0.0003	0.2897	0.0007
R14	0.2833	0.0007	0.3797	0.0019	E02	0.2039	0.0002	0.3467	0.0014
R15	0.3266	0.0011	0.4064	0.0024	E03	0.3292	0.0012	0.4987	0.0049
R16	0.2280	0.0003	0.3052	0.0009	E04	0.3743	0.0018	0.4764	0.0042
R17	0.3543	0.0015	0.4580	0.0036	E05	0.4698	0.0040	0.5986	0.0096
R18	0.5061	0.0052	0.6164	0.0108	E06	0.4138	0.0026	0.5561	0.0073
R19	0.3227	0.0011	0.3886	0.0021	E07	0.3930	0.0021	0.5408	0.0066
R20	0.2731	0.0006	0.3495	0.0014	E08	0.3732	0.0018	0.5164	0.0056
D01	0.4531	0.0035	0.6344	0.0121	E09	0.4762	0.0042	0.6053	0.0101
D02	0.5312	0.0062	0.6122	0.0105	E10	0.3999	0.0023	0.5852	0.0088
D03	0.2936	0.0008	0.4386	0.0031	E11	0.4122	0.0025	0.5264	0.0060
D04	0.3812	0.0019	0.4754	0.0041	E12	0.4101	0.0025	0.5258	0.0059
D05	0.3077	0.0009	0.4176	0.0026	E13	0.3572	0.0015	0.4314	0.0030
D06	0.3691	0.0017	0.4650	0.0038	E14	0.2892	0.0007	0.3822	0.0019
D07	0.4610	0.0037	0.5597	0.0075	E15	0.4541	0.0035	0.5635	0.0077

- IFS mode, which uses intuitionistic fuzzy sets without considering confidence levels
- IZN mode, which incorporates expert confidence through Z-number theory

A direct comparison between these two modes reveals a consistent reduction in the estimated probabilities when using IZN. For instance, BE R01 has a prior failure probability of 0.0111 in IFS, whereas in IZN, the probability drops to 0.0033. This pattern is evident across the dataset and demonstrates how including expert confidence levels leads to more conservative (and arguably more realistic) assessments. By comparing the two modes, the model clearly illustrates the impact of confidence-weighted expert input on risk estimation, emphasizing the added value of using IZN in high-uncertainty environments.

These BE-level probabilities were then propagated through the barrier fault trees using logical gate relationships defined in Equations (30–32), resulting in prior failure probabilities for the four primary safety barriers: RPB, DPB, IPB, and EPB. Following this, the prior probabilities of system-level consequences were derived via the event tree structure using Equation (33). The consequences are categorized into safe, near miss, Mishap, incident, and accident, following established classifications in the literature [38].

**Fig 6:**
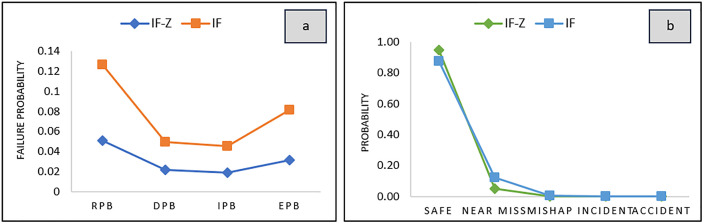
Prior Probabilities of (a) Barrier Failure and (b) Consequence.

The results are summarized in Table 6, which presents the barrier failure probabilities and consequence probabilities in both IFS and IZN modes. For example, the Release Prevention Barrier (RPB) exhibited the highest failure probability under IFS (0.1270), which was reduced significantly in IZN mode. This difference is clearly illustrated in Fig 6, which provides a visual comparison of outcomes between the two modeling approaches. Overall, these results confirm that the IZN-based approach yields more stable and realistic assessments, offering a practical advantage in high-uncertainty risk environments.

**Table 6:** The prior probability of barrier failure and consequences in two modes of IFS and IZN.

		IZN	IFS
Barrier’s failure	RPB	0.0511	0.1270
	DPB	0.0215	0.0495
	IPB	0.0189	0.0450
	EPB	0.0314	0.0812
Consequences	Safe	0.9489	0.8730
	Near miss	0.0500	0.1208
	Mishap	0.0011	0.0060
	Incident	0.2$\;\times {\text{1}\text{0}}^{-\text{4}}$	${0.26\times 10}^{-3}$
	Accident	0.1 $\times {\text{1}\text{0}}^{-\text{5}}$	${0.23\times 10}^{-4}$

The computed prior probabilities generated in this section serve as foundational inputs for the dynamic risk analysis and Bayesian updating presented in the following section.

### 4.3. Updating failure probability

To assess how risk evolves over time, we collected monthly data on abnormal events involving gas leakage from LPG spherical tanks, as shown in Table 7. This cumulative dataset—spanning 12 months—includes five consequence categories: Safe, Near Miss, Mishap, Incident, and Accident. These values form the basis for calculating likelihood probabilities of safety barrier failures each month, presented in Table 8. For example, the RPB showed an increasing likelihood trend, reaching 0.7659 in month 12, indicating its deteriorating performance over time. Similarly, DPB and IPB showed moderate failure likelihoods of 0.4197 and 0.4074 respectively in the same period. The EPB, although generally lower, peaked at 0.1429 in month 8—highlighting a moment of elevated vulnerability.

**Table 7:** The cumulative number of abnormal events that have occurred during the first 12 months.

Month	Safe	Near miss	Mishap	Incident	Accident
1	5	5	2	1	0
2	9	13	4	3	0
3	15	23	7	4	0
4	23	28	10	6	0
5	31	34	14	8	1
6	35	48	22	13	2
7	47	56	27	14	2
8	51	71	31	18	3
9	55	83	39	22	3
10	55	91	42	24	3
11	58	102	45	28	4
12	59	112	48	29	4

**Table 8:** Likelihood probability of each barrier.

	RPB	DPB	IPB	EPB
1	0.6154	0.3750	0.3333	0.00
2	0.6897	0.3500	0.43	0.00
3	0.6939	0.3235	0.364	0.00
4	0.6567	0.3636	0.3750	0.00
5	0.6477	0.4035	0.3913	0.1111
6	0.7083	0.4353	0.4054	0.1333
7	0.6781	0.40	0.3721	0.1250
8	0.7069	0.42276	0.4038	0.1429
9	0.7277	0.43537	0.3906	0.1200
10	0.7442	0.43125	0.3913	0.1111
11	0.7553	0.43017	0.4156	0.1250
12	0.7659	0.4197	0.4074	0.1212

By applying Bayes’ theorem (Eq. 43), we integrated these monthly likelihoods with the prior probabilities (from Table 6) to derive the posterior probabilities of barrier failures, shown in Table 9. The IZN-based results consistently yielded lower posterior values, suggesting more conservative risk estimation when accounting for expert confidence. In the twelfth month, for example, the failure probability of RPB was 0.3225 under IFS, whereas it decreased to 0.1498 using IZN. A similar pattern was observed for the IPB, with probabilities of 0.0314 in IFS and 0.0131 in IZN. These differences highlight the impact of accounting for uncertainty in expert judgment through intuitionistic Z-numbers, resulting in more reliable and realistic assessments of system vulnerability.

**Table 9:** Posterior probability of barrier failure in two states of calculations, IFS and IZN.

	RPB		DPB		IPB		EPB	
	IZN	IFS	IZN	IFS	IZN	IFS	IZN	IFS
1	0.0793	0.1889	0.0130	0.0303	0.0095	0.0230	0.00	0.00
2	0.1069	0.2444	0.0117	0.0273	0.0142	0.0341	0.00	0.00
3	0.1088	0.2480	0.0104	0.0243	0.0109	0.0262	0.00	0.00
4	0.0934	0.2178	0.0124	0.0289	0.0114	0.0275	0.00	0.00
5	0.0901	0.2111	0.0147	0.0340	0.0122	0.0294	0.0040	0.0109
6	0.1157	0.2611	0.0167	0.0386	0.0130	0.0311	0.0050	0.0134
7	0.1019	0.2346	0.0166	0.0384	0.0113	0.0272	0.0046	0.0125
8	0.1150	0.2598	0.0159	0.0367	0.0129	0.0309	0.0054	0.0145
9	0.1259	0.2800	0.0167	0.0386	0.0122	0.0293	0.0044	0.0119
10	0.1355	0.2974	0.0164	0.0380	0.0122	0.0294	0.0040	0.0109
11	0.1425	0.3099	0.0163	0.0378	0.0135	0.0324	0.0046	0.0125
12	0.1498	0.3225	0.0157	0.0363	0.0131	0.0314	0.0045	0.0120

**Fig 7:**
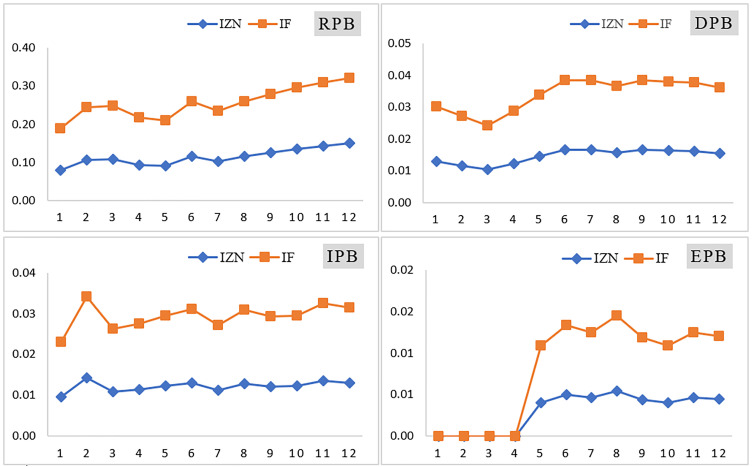
Temporal pattern of the posterior probability of barrier failures in the 12 months (The vertical axis is the probability value, and the horizontal axis is the period).

Fig 7, shows the temporal trend of posterior failure probabilities across the 12-month period. The observed increase in failure likelihoods—particularly for RPB and DPB—suggests progressive system degradation or the declining effectiveness of preventive measures over time.

The posterior probabilities were then propagated through the event tree model (Fig 4) using eq. 33 to estimate the likelihood of various consequence levels over time. as presented in Table 10, these estimations were calculated under both IFS and IZN modes. The results indicate a gradual decline in the probability of safe outcomes—from 0.9207 in month 1 to 0.8500 in month 12 under the IZN approach, and from 0.8111 to 0.7026 under IFS—reflecting an overall increase in operational risk as time progressed. Concurrently, the probability of experiencing a near-miss event showed a marked increase, reaching 0.3108 in month 12 under IFS and 0.1402 under IZN, suggesting growing exposure to safety-critical conditions. Although the probabilities of incidents and accidents remained relatively low in absolute terms, they exhibited an upward trend, nearly doubling over the course of the 12-month period. For instance, the probability of an accident under IZN, initially zero, rose to 1.45 × 10 ⁻ ⁷ by the end of the study period, emphasizing the importance of continuous monitoring and dynamic risk updating in high-hazard industrial environments.

**Table 10:** Posterior probability of consequences in two states of calculations, IFS and IZN.

	Safe		Near miss		Mishap		Incident		Accident	
	IZN	IFS	IZN	IFS	IZN	IFS	IZN	IFS	IZN	IFS
1	0.9207	0.8111	0.0783	0.1831	0.0010	0.0056	9.852E-06	0.00013	0.00	0.00
2	0.8931	0.7556	0.1057	0.2377	0.0012	0.0064	1.781E-05	0.00023	0.00	0.00
3	0.8912	0.7520	0.1077	0.2420	0.0011	0.0059	1.232E-05	0.00016	0.00	0.00
4	0.9066	0.7822	0.0923	0.2115	0.0011	0.0061	1.324E-05	0.00017	0.00	0.00
5	0.9099	0.7889	0.0888	0.2039	0.0013	0.0070	1.608E-05	0.00021	6.525E-08	2.306E-06
6	0.8843	0.7389	0.1138	0.2511	0.0019	0.0098	2.486E-05	0.00031	1.241E-07	4.205E-06
7	0.8981	0.7654	0.1002	0.2256	0.0017	0.0088	1.900E-05	0.00024	8.809E-08	3.053E-06
8	0.8850	0.7402	0.1132	0.2503	0.0018	0.0092	2.334E-05	0.00029	1.262E-07	4.282E-06
9	0.8741	0.7200	0.1238	0.2692	0.0021	0.0105	2.547E-05	0.00031	1.127E-07	3.772E-06
10	0.8645	0.7026	0.1333	0.2861	0.0022	0.0110	2.705E-05	0.00033	1.097E-07	3.627E-06
11	0.8575	0.6901	0.1402	0.2982	0.0023	0.0113	3.129E-05	0.00038	1.451E-07	4.735E-06
12	0.8502	0.6775	0.1475	0.3108	0.0023	0.0113	3.050E-05	0.00036	1.365E-07	4.420E-06

### 4.4. Sensitivity and Validation Analysis of the Methodology

A sensitivity analysis was conducted to evaluate and validate the performance of the proposed IZN-based method in comparison to the traditional IFS approach, using the posterior and prior probabilities presented in Tables 9 and 10. This analysis highlights how the calculated posterior probabilities respond to changes in prior inputs under both fuzzy environments. As discussed in Section 4.3, the degree of variation between prior and posterior probabilities in IZN and IFS modes provides insight into each method’s responsiveness to real-world event data. The results indicate that IZN offers a more stable and conservative adjustment, which is critical for identifying worst-case scenarios and supporting robust decision-making under uncertainty.

## 5. Discussion

This study performed a dynamic risk assessment using two calculation modes: Intuitionistic Fuzzy Sets (IFS) and Intuitionistic Z-Numbers (IZN). The objective was to evaluate the effect of incorporating expert confidence levels into the final probability estimates. By comparing the outcomes from both approaches, the influence of confidence-weighted inputs on the overall results can be clearly observed, offering valuable insights into the role of expert reliability in risk quantification.

As illustrated in Fig 6a, the prior failure probabilities of the safety barriers do not follow a uniform or gradual increasing trend across barriers; instead, they exhibit a non-linear pattern. This indicates that the failure probability of one barrier cannot be reliably estimated based on the preceding one. These findings are consistent with those reported in previous studies [17,38], highlighting how the frequency and nature of abnormal events reported within an industrial setting can significantly shape the distribution pattern of failure probabilities.

**Fig 8:**
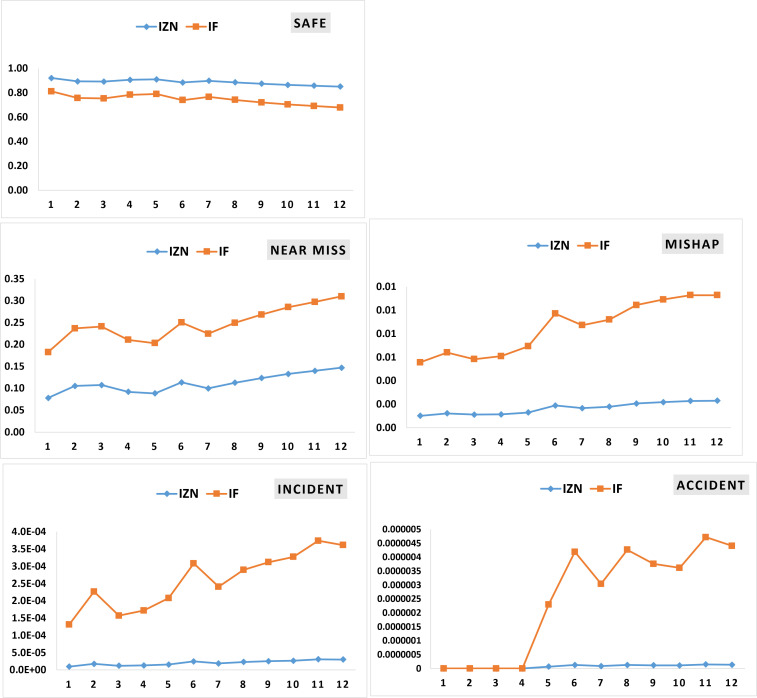
Temporal pattern of the posterior probability of consequences in the 12 months (The vertical axis is the probability value, and the horizontal axis is the period).

Fig 8 clearly illustrates the impact of incorporating expert confidence levels on the estimation of prior probabilities. The comparison highlights how accounting for confidence levels influences the final probability values, underscoring the importance of including this factor in the analysis. Similar findings were reported by Yazdi et al. (2019) [35] who employed both traditional fuzzy combination methods and Z-number techniques, further supporting the results of the present study.

In contrast, the prior probabilities of consequences show minimal differences between the IFS and IZN modes, primarily due to the low magnitude of the calculated values. Additionally, the probability associated with the “safe” outcome is substantially higher than that of the adverse consequences, resulting in reduced variation and a smaller visual gap in the corresponding graph. However, Table 6 offers a more detailed comparison by presenting the specific prior probability values under both modes, providing a clearer understanding of the differences between the IFS and IZN approaches.

A comparison of Figs 7 and 8 reveals a general trend in which the posterior probability of consequence occurrence over a 12-month period aligns with the posterior probability of barrier failure. However, this correlation does not hold consistently across all months. Such discrepancies may be attributed to factors such as a low number of recorded abnormal events or a low prior probability of the Basic Events (BEs) associated with a given barrier. These conditions can reduce the sensitivity of the analysis and influence the patterns observed in the resulting charts.

The posterior probability trend for barrier failure, as shown in Fig 7, indicates a gradual increase over the 12 months. This trend may be attributed to factors such as equipment aging, wear and tear, or a decline in employee vigilance regarding inspections and maintenance activities over time. Similarly, the consequence diagrams in Fig 8 show rising probabilities for near misses, mishaps, incidents, and accidents. In the IFS-based analysis, the probabilities of incidents and accidents increase more steeply than those of near misses and mishaps. In contrast, under the IZN-based approach, the steepest increase is observed for near misses. This difference is driven by the integration of Z-number theory with intuitionistic fuzzy logic, combined with the number of abnormal events recorded monthly as primary input data.

Figs 7 and 8 indicate that, in IZN mode, the slope of increase is more gradual compared to the IFS mode, and the intensity of fluctuations is noticeably lower. This observation is significant, as incorporating Z-numbers into dynamic risk assessment can influence risk prioritization. By providing a more stable and confidence-weighted evaluation, the use of Z-numbers enhances the efficiency of resource and equipment allocation for accident prevention, ultimately supporting more effective risk management strategies.

Given the nature of the main event—gas leakage from a spherical tank—the most critical barrier for preventing subsequent consequences is the Release Prevention Barrier (RPB). Preventing the initial release of gas effectively eliminates the formation of a hazardous gas cloud, thereby avoiding potential explosions and escalation. Focusing risk assessment efforts on the failure analysis of this primary barrier offers a highly efficient strategy for mitigating severe outcomes. This targeted approach enables optimal risk reduction with minimal resource expenditure, making it a cost-effective solution in safety management.

The findings of this research are broadly consistent with recent studies that have adopted advanced decision-making or AI-based techniques to address safety and risk-related problems in different engineering domains. For instance, fuzzy DEMATEL and COPRAS approaches have been applied to analyze railway accident risks [13,48], while hybrid AI-driven models have been developed to predict workplace accidents and support proactive control strategies in large-scale projects [49–51]. Comparable methodologies have also been introduced in supply chain management and transportation safety contexts [14,52,53]. Taken together, these works point to a wider trend toward embedding intelligent systems in risk assessment, which resonates with and reinforces the implications of our results.

A key advantage of the proposed method is its ability to be dynamically updated with new information regarding barrier failures. This feature allows for real-time updates of consequence probabilities, enabling timely and informed responses to emerging critical situations. Similar comprehensive studies in this domain [3,46] have arrived at comparable conclusions. Overall, the findings support the effectiveness of the proposed framework in adapting risk assessment based on the evolving behavior of the system. By integrating this dynamic approach, organizations can One limitation of this study was the inability to perform more precise updates due to the lack of detailed documentation on high-consequence events, a challenge also noted in previous studies using the SHIPP methodology [17,38,47]. Additionally, the focus on a specific case study may limit the generalizability of the findings. As with similar research, reliance on expert opinions from diverse backgrounds could introduce inherent bias, despite efforts to standardize and weight the inputs.

## 6. Conclusion

This study successfully developed a novel, dynamic risk assessment approach by integrating Intuitionistic Z-Numbers (IZNs) into the System Hazard Identification, Prediction, and Prevention (SHIPP) methodology. This integration effectively addresses the inherent uncertainty in expert judgments and provides a more robust framework for estimating the probability of basic events. The results of a case study on LPG spherical tanks demonstrate that incorporating expert confidence levels through the IZN method significantly reduces uncertainty in failure probability estimates compared to traditional Intuitionistic Fuzzy Set (IFS) methods.

The practical application of this approach showed that both prior and posterior probabilities of barrier failure, calculated using IZN, were consistently lower and more realistic than those obtained with IFS. For instance, the posterior probability of a near-miss consequence in month 12 was 0.1475 with IZN, compared to 0.3108 with IFS. These findings highlight the enhanced reliability and sensitivity of the IZN-SHIPP approach in tracking dynamic changes in system behavior, ultimately improving the accuracy of predicting abnormal events and supporting more informed risk management decisions. Future research could extend this framework to multi-barrier and multi-scenario environments, incorporate real-time sensor data for live risk updates, or explore its applicability in other domains such as transportation and healthcare.

## Supporting information

S1 Table Containing the following: S1A Table, experts’ opinion about BEs prior p robability. S1B Table, experts’ confidence about themselves opinion.
(DOCX)

S1 Appendix An example of Aggregation computation for R10 Fig 6. a: Prior probability of barrier failure b: Prior probability of consequence. (DOCX)
