# Supplementary material for: A novel approach to managing uncertainty in risk assessment using integrated z-numbers and intuitionistic fuzzy sets: A case study on LPG spherical tanks
Source: PLoS One. 2026 Feb 26;21(2):e0338798. doi: 10.1371/journal.pone.0338798 (PMC12944727; doi:10.1371/journal.pone.0338798)
Supplement: S1 Table — robability. S1B Table, experts’ confidence about themselves opinion. (DOCX) [file pone.0338798.s001.docx]

**Supporting Information**

**S1 Table**

S1A Table, experts’ opinion about BEs prior probability

| BEs | E1 | E2 | E3 | E4 | E5 | E6 | BEs | E1 | E2 | E3 | E4 | E5 | E6 |
| --- | --- | --- | --- | --- | --- | --- | --- | --- | --- | --- | --- | --- | --- |
| R01 | ML | M | MH | MH | MH | MH | **D08** | H | M | ML | MH | M | L |
| R02 | M | MH | M | H | MH | M | **IP01** | ML | ML | ML | M | ML | L |
| R03 | MH | M | M | H | M | M | **IP02** | L | M | ML | M | M | ML |
| R04 | H | ML | L | VL | VH | H | **IP03** | M | MH | L | L | L | ML |
| R05 | M | VH | VL | H | M | L | **IP04** | M | ML | M | ML | VL | M |
| R06 | ML | ML | H | H | ML | L | **IP05** | ML | H | H | ML | VL | M |
| R07 | M | MH | M | M | H | M | **IP06** | VL | ML | H | M | VH | VL |
| R08 | L | ML | H | MH | M | L | **IP07** | M | VL | MH | MH | M | MH |
| R09 | MH | ML | H | L | H | H | **IP08** | H | H | VL | VH | ML | L |
| R10 | M | VL | L | M | VH | H | **IP09** | ML | ML | M | M | VH | VL |
| R11 | L | H | VL | ML | VH | H | **IP10** | ML | MH | ML | L | MH | M |
| R12 | VH | L | L | M | VH | VH | **IP11** | L | M | ML | M | VH | MH |
| R13 | VL | M | L | M | VH | ML | **E01** | L | L | M | MH | ML | L |
| R14 | ML | L | L | ML | VH | MH | **E02** | ML | VL | L | H | ML | M |
| R15 | ML | M | M | L | VH | L | **E03** | ML | H | H | ML | VL | M |
| R16 | ML | M | ML | L | M | L | **E04** | M | M | H | M | ML | L |
| R17 | L | M | M | M | VH | L | **E05** | L | H | M | VL | VH | MH |
| R18 | M | MH | MH | VL | VH | M | **E06** | MH | ML | H | L | M | MH |
| R19 | L | M | VL | VL | MH | H | **E07** | M | VL | L | M | VH | H |
| R20 | L | L | MH | M | M | VL | **E08** | M | L | M | M | VH | M |
| D01 | MH | MH | H | ML | MH | VL | **E09** | ML | H | ML | M | VH | MH |
| D02 | VL | VL | VH | M | VH | VH | **E10** | L | H | M | ML | MH | H |
| D03 | L | ML | L | M | VH | MH | **E11** | M | L | MH | M | MH | M |
| D04 | ML | ML | M | MH | H | VL | **E12** | H | ML | MH | M | M | ML |
| D05 | MH | L | L | ML | M | MH | **E13** | ML | H | H | L | ML | VL |
| D06 | M | L | ML | ML | VH | MH | **E14** | M | M | ML | ML | M | L |
| D07 | H | L | M | M | VH | ML | **E15** | M | M | MH | M | H | VL |

S1B Table, experts’ confidence about themselves opinion

| BEs | E1 | E2 | E3 | E4 | E5 | E6 | BEs | E1 | E2 | E3 | E4 | E5 | E6 |
| --- | --- | --- | --- | --- | --- | --- | --- | --- | --- | --- | --- | --- | --- |
| R01 | RS | RS | RS | S | S | S | **D08** | S | S | S | VS | RS | S |
| R02 | RS | VS | VS | S | S | RS | **IP01** | S | RS | S | S | S | VS |
| R03 | S | RS | RS | S | S | S | **IP02** | VS | VS | VS | VS | VS | VS |
| R04 | S | S | S | S | VS | S | **IP03** | S | VS | S | S | S | VS |
| R05 | RS | VS | S | S | RS | S | **IP04** | VS | S | S | VS | S | VS |
| R06 | VS | RS | S | S | VS | VS | **IP05** | S | RS | RS | S | S | RS |
| R07 | VS | S | S | VS | VS | S | **IP06** | VS | RS | S | VS | RS | RS |
| R08 | VS | S | S | S | VS | VS | **IP07** | S | S | RS | VS | VS | VS |
| R09 | S | RS | S | NS | RS | S | **IP08** | VS | S | S | S | S | S |
| R10 | RS | VS | RS | RS | VS | S | **IP09** | RS | S | S | RS | RS | S |
| R11 | S | S | S | S | S | VS | **IP10** | RS | S | VS | VS | S | RS |
| R12 | S | RS | S | RS | RS | RS | **IP11** | S | S | S | S | RS | S |
| R13 | S | S | S | S | S | VS | **E01** | VS | S | S | RS | VS | S |
| R14 | S | S | RS | RS | RS | VS | **E02** | VS | RS | VS | NS | RS | NS |
| R15 | S | S | S | S | VS | S | **E03** | S | RS | RS | S | S | RS |
| R16 | S | S | RS | RS | S | S | **E04** | VS | RS | S | VS | RS | RS |
| R17 | S | S | RS | S | VS | RS | **E05** | S | S | RS | S | S | VS |
| R18 | VS | S | S | VS | VS | S | **E06** | S | RS | S | NS | RS | S |
| R19 | S | S | VS | S | VS | S | **E07** | RS | VS | RS | RS | VS | S |
| R20 | RS | VS | S | S | S | VS | **E08** | S | S | S | RS | S | RS |
| D01 | S | S | RS | VS | RS | S | **E09** | S | VS | S | VS | RS | S |
| D02 | RS | S | S | VS | VS | VS | **E10** | RS | S | S | S | S | NS |
| D03 | S | VS | VS | NS | S | RS | **E11** | S | S | S | VS | RS | VS |
| D04 | S | S | S | S | VS | S | **E12** | S | NS | S | VS | S | RS |
| D05 | S | RS | S | S | S | RS | **E13** | VS | S | VS | VS | S | S |
| D06 | S | RS | VS | NS | S | VS | **E14** | S | RS | S | S | VS | VS |
| D07 | VS | S | VS | S | S | RS | **E15** | VS | S | VS | RS | S | VS |
