# Supplementary material for: A novel approach to managing uncertainty in risk assessment using integrated z-numbers and intuitionistic fuzzy sets: A case study on LPG spherical tanks
Source: PLoS One. 2026 Feb 26;21(2):e0338798. doi: 10.1371/journal.pone.0338798 (PMC12944727; doi:10.1371/journal.pone.0338798)
Supplement: S1 Appendix — (DOCX) [file pone.0338798.s002.docx]

***S1 Appendix. an example of*** ***Aggregation computation for R10***

|  | A | B | Z = (A, B) | |
| --- | --- | --- | --- | --- |
| Expert 1 | M | RS | [(0.35, 0.50, 0.65; 0.32, 0.50, 0.68), (0.20, 0.40, 0.60; 0.10, 0.40, 0.70)] | |
| Expert 2 | VL | VS | [(0.00, 0.04, 0.08; 0.00, 0.04, 0.08), (0.60, 0.80, 1.00; 0.60, 0.80, 1.00)] | |
| Expert 3 | L | RS | [(0.07, 0.13, 0.19; 0.06, 0.13, 0.20), (0.20, 0.40, 0.60; 0.10, 0.40, 0.70)] | |
| Expert 4 | M | RS | [(0.35, 0.50, 0.65; 0.32, 0.50, 0.68), (0.20, 0.40, 0.60; 0.10, 0.40, 0.70)] | |
| Expert 5 | VH | VS | [(0.92, 0.96, 1.00; 0.92, 0.96, 1.00), (0.60, 0.80, 1.00; 0.60, 0.80, 1.00)] | |
| Expert 6 | H | S | [(0.81, 0.87, 0.93; 0.79, 0.87, 0.95), (0.40, 0.60, 0.80; 0.30, 0.60, 0.90)] | |
| Regular fuzzy (Eq. 18 & 19) | Expert 1 | $S_{B}=\frac{1}{3}\left[ \frac{\left( 0.7-0.1 \right)\left( 0.4-2\left( 0.7 \right)-2\left( 0.1 \right) \right)+\left( 0.6-0.2 \right)\left( 0.2+0.4+0.6 \right)+3\left( {0.7}^{2}-{0.1}^{2} \right)}{0.7-0.1+0.6-0.2} \right]=0.4$  (0.35$\times\sqrt{0.4}$, 0.50$\times\sqrt{0.4}$, 0.65$\times\sqrt{0.4}$; 0.32$\times\sqrt{0.4}$, 0.50$\times\sqrt{0.4}$, 0.68$\times\sqrt{0.4}$)  (0.2213, 0.3162, 0.4111; 0.2023, 0.3162, 0.430) | | |
|  | Expert 2 | (0, 0.0357, 0.0715; 0, 0.0357, 0.0715) | | |
|  | Expert 3 | (0.0442, 0.0822, 0.120; 0.0379, 0.0822, 0.1264) | | |
|  | Expert 4 | (0.2213, 0.3162, 0.4111; 0.2023, 0.3162, 0.430) | | |
|  | Expert 5 | (0.8228, 0.8586, 0.8944; 0.8228, 0.8586, 0.8944) | | |
|  | Expert 6 | (0.6274, 0.6739, 0.7203; 0.6119, 0.6739, 0.7358) | | |
| Using Eq.20 & 21 | S ($E_{1}\&E_{2}$) | 0.54 | | ${EV}_{1}=\frac{0.2213+0.2023+4\left( 0.3162 \right)+(0.4111+0.430)}{8}=$0.1707  ${EV}_{2}=\frac{0+0+4\left( 0.0357 \right)+(0.0715+0.0715)}{8}=$0.3162  ${EV}_{1}\leq{EV}_{2}\to\frac{{EV}_{1}}{{EV}_{2}}=0.54$ |
|  | S ($E_{1}\&E_{3}$) | 0.3711 | |  |
|  | S ($E_{1}\&E_{4}$) | 0.3030 | |  |
|  | S ($E_{1}\&E_{5}$) | 0.3030 | |  |
|  | S ($E_{1}\&E_{6}$) | 0.3030 | |  |
| Using Eq. 23 | AA ($E_{1}$) | 0.3640 | | $AA\left( E_{i} \right)=\frac{1}{n-1}\sum_{\begin{aligned} j=j \\ j\neq1 \end{aligned}}^{n} S_{ij}\left( \tilde{A}_{i}, \tilde{A}_{j} \right) i=1, 2, \ldots, n$  $=\frac{1}{6-1}\left( 0.54+0.3711+0.3030+0.3030+0.3030 \right)=0.3640$ |
|  | AA ($E_{2}$) | 0.5821 | |  |
|  | AA ($E_{3}$) | 0.7015 | |  |
|  | AA ($E_{4}$) | 0.7361 | |  |
|  | AA ($E_{5}$) | 0.7361 | |  |
|  | AA ($E_{6}$) | 0.7361 | |  |
| Using Eq. 24 | RA ($E_{1}$) | 0.0944 | | $RA\left( E_{i} \right)=\frac{AA\left( E_{i} \right)}{\sum_{i=1}^{n} AA(E_{i})} i=1, 2, \ldots, n$  $=\frac{0.3640}{0.3640+0.5821+0.7015+0.7313+0.7313+0.7313}=0.0944$ |
|  | RA ($E_{2}$) | 0.1509 | |  |
|  | RA ($E_{3}$) | 0.1819 | |  |
|  | RA ($E_{4}$) | 0.1908 | |  |
|  | RA ($E_{5}$) | 0.1908 | |  |
|  | RA ($E_{6}$) | 0.1908 | |  |
| Using Eq. 25 & 26 | C ($E_{1}$) | 0.1454 | | $WF\left( E_{1} \right)=\frac{WS\left( E_{1} \right)}{\sum_{i=1}^{n} WS\left( E_{i} \right)}\underset{\to}{Table 4} \frac{14}{11+11+8+10+9+7}=0.1964$  $C\left( E_{i} \right)=\beta⨀ WF\left( E_{i} \right)+\left( 1-\beta\right) ⨀ RA\left( E_{i} \right)$  $=0.5\times0.1964+0.5\times0.8036=0.1486$ |
|  | C ($E_{2}$) | 0.1736 | |  |
|  | C ($E_{3}$) | 0.1623 | |  |
|  | C ($E_{4}$) | 0.1847 | |  |
|  | C ($E_{5}$) | 0.1758 | |  |
|  | C ($E_{6}$) | 0.1579 | |  |
| Using Eq.27 | Aggregation for R10 | | | $\tilde{P}_{j}=\sum_{i=1}^{n} C\left( E_{i} \right)\otimes\tilde{P}_{ij}, j=1, 2, \ldots, n.$  $=0.1454\otimes\left( 0.2213, 0.3162, 0.4111; 0.2023, 0.3162, 0.430 \right) \bigoplus0.1736\otimes\left( 0, 0.0357, 0.0715; 0, 0.0357, 0.0715 \right) \bigoplus0.1623\otimes\left( 0.0442, 0.0822, 0.120; 0.0379, 0.0822, 0.1264 \right) \bigoplus0.1847\otimes\left( 0.2213, 0.3162, 0.4111; 0.2023, 0.3162, 0.430 \right)\bigoplus0.1758\otimes(0.8228, 0.8586, 0.8944; 0.8228, 0.8586, 0.8944)\bigoplus0.1579\otimes(0.6274, 0.6739, 0.7203; 0.6119, 0.6739, 0.7358)$ |
